# Successful adherence and retention to daily monitoring of physical activity: Lessons learned

**DOI:** 10.1371/journal.pone.0199838

**Published:** 2018-09-20

**Authors:** Xiaomeng Xu, Samantha Tupy, Stephen Robertson, Ashley L. Miller, Danielle Correll, Rick Tivis, Claudio R. Nigg

**Affiliations:** 1 Department of Psychology, Idaho State University, Pocatello, Idaho, United States of America; 2 Department of Psychology, University of Oregon, Eugene, Oregon, United State of America; 3 Department of Psychology, University of Colorado Colorado Springs, Colorado Springs, Colorado, United States of America; 4 Idaho Center for Health Research, Idaho State University, Meridian, Idaho, United States of America; 5 Boise VA Medical Center of Excellence for Primary Care Education, Boise, Idaho, United States of America; 6 Office of Public Health Studies, University of Hawai’i at Manoa, Honolulu, Hawaii, United States of America; West Chester University of Pennsylvania, UNITED STATES

## Abstract

Research utilizing repeated-measures such as daily assessments with self-report and/or objective measures [e.g., physical activity (PA) monitors] are important in understanding health behaviors and informing practice and policy. However, studies that utilize daily assessment often encounter issues with attrition and non-compliance. The current research yielded high levels of retention and adherence with both self-report and objective daily measures. The purpose of this paper is to highlight and discuss strategies utilized in maximizing retention, minimizing missing data, and some lessons learned from the research experience. Fifty community participants took part in a 4-week study utilizing both daily self-report questionnaires and daily use of PA monitors (Fitbit One^™^). This study focused on typical daily PA and was not an intervention study (e.g., participants were not randomized nor asked to change their PA behavior). Participants completed the study in two waves (wave 1 *n* = 10, wave 2 *n* = 40). The research team utilized several retention strategies including automating the data collection process, a prorated incentive structure, having a dedicated and responsive study staff, and utilizing the 2-wave process to optimize data collection during the 2^nd^ wave. The study had 100% retention and generally positive anonymous feedback post-study. Overall, participants completed the vast majority of daily surveys (97%) and wore their Fitbits (for at least part of the day) on almost all days (99.57%) of the study, although there were individual differences. The strategies discussed and lessons learned may be useful to other researchers using daily measurements for whom adherence and retention are important issues. Future research employing these strategies in different populations, with different measurements, and for longer durations is warranted to determine generalizability.

## Introduction

Engaging in regular physical activity (PA) is associated with better physical health [1–3] and improved psychological functioning [4, 5] relative to those who are physically inactive. Recently, technology has been introduced into consumer markets that allow individuals to monitor PA levels (e.g., pedometers) and this technology often claims to help increase engagement in PA. Researchers also utilize this technology in assessment of PA and in studies evaluating the claims that these products encourage increased engagement in PA, typically finding a positive effect of PA monitors on PA (e.g., [6, 7]).

PA tracking technology can be used concurrently with daily diary methods to provide a greater wealth of information on PA and PA tracking technology use (e.g., [8]). Daily diary methods involve requiring participants to record information daily at predetermined intervals and can be used to track changes in psychological, behavioral, and health related phenomena [9]. However, published reports of both PA tracking technology and daily diary methods have documented problems with participant retention and protocol adherence [7, 8,10, 11]. As such, it is important to understand the factors that promote retention and adherence to allow researchers to more efficiently and effectively conduct both daily diary and PA tracking studies.

Daily diary studies are advantageous as they document experiences at set time points longitudinally and allow for data collection over an extended period of time in naturally occurring contexts [9]. However, the increased response frequency provides additional opportunities for non-adherence and attrition. For example, Senturia and colleagues (1998) reported a study in which participants attended multiple interviews and recorded daily diary entries [11]. At the 9-month follow-up, 89% of the participants had been retained; however, only 42% completed the daily diary. A more recent study also documented a similar trend. Specifically, Gautreau et al. (2015) asked participants to record daily diary entries across 14 days and attend a follow-up session 14 days after the daily diary portion of the study concluded [12]. Results indicated that despite 97.6% of the participants attending the final laboratory session, participants only completed 75.2% to 84.1% of the daily diary entries across the daily diary portion of the study. Thus, even if retention strategies are effective at encouraging multiple laboratory visits, they may not adequately influence daily diary compliance.

Unfortunately, daily diary studies are not alone in encountering these problems. Research using PA monitors (e.g., [10, 13, 14]) likewise experience issues with attrition. For instance, studies have shown retention rates of 56% for a 10-week study [15], 61% for a 12-week study [13], and 72% for a 3-month study [7]. Characterizing methods to enhance retention in PA monitor studies, regardless of population utilized or duration of the study, is an important step in improving the intervention’s efficacy.

Technology that permits recording of objective PA data may be useful when paired with daily diary methodology for reducing reliance on participants’ responses; however, using these methods concurrently introduces a variety of opportunities for non-adherence. Fukuoka and colleagues (2015) investigated factors that influence adherence and retention in a study that utilized both PA monitors and daily diaries across a 3-week assessment period in which participants were asked to wear a pedometer daily and complete daily diary entries via a mobile phone application [8]. In this study, researchers defined adherence as wearing a pedometer and completing the daily diaries 80% of the time during the study. Thirty-four percent of participants did not adhere to this aspect of the study. Of the 34.0% who did not show adherence, 50.9% failed to wear the pedometer at least 8 hours per day and/or complete the daily diaries for at least 80% of the assessment phase. Researchers compared individuals who successfully completed the study to those who did not and found that individuals who were not successful tended to be younger, earned less money, self-reported being less healthy, had restricted access to transportation, and were generally unfamiliar with using pedometers. As such, these authors documented that a variety of problems can occur when using daily diary and PA monitors—such as trouble with technology, not being comfortable using the technology, and participant characteristics. It is possible that designing components of an intervention to overcome these obstacles will result in better retention and adherence.

The current study seeks to add to the extant literature on adherence and retention in studies that use daily diary techniques and PA monitoring. In this study, we utilized the Fitbit One^™^ to track PA. Fitbit is a popular brand of PA monitors and offers several models that have been utilized in research. Studies have demonstrated that the Fitbit is an effective tool to collect daily PA intervention data [16, 17], and that the Fitbit One (a device worn at the hip) is a valid and reliable device (e.g., [18, 19]). Therefore, the Fitbit has potential for use in behavioral health research to characterize and increase PA. While our overall research project included hypotheses about the relation between PA and other (e.g., psychological) factors, this paper focuses on adherence and retention in a 4-week study that utilized daily Fitbit One monitoring and daily self-report questionnaires. Specifically, we discuss our strategies and the lessons we learned (e.g., what we would do differently) with the goal of helping other researchers enhance retention and adherence in their studies.

## Methods and materials

The Institutional Review Board/Human Subjects Committee of Idaho State University approved this research.

### Participants

A total of 50 community adults participated in this 4-week research study. Recruitment of participants from the Southeast Idaho community involved flyers, announcements in listservs, and word of mouth. Interested participants contacted the study personnel through a designated email at which point a phone screening was conducted to assess eligibility. To be eligible for the study, individuals had to be 18 years or older, English speaking, report absence of medical issues that would prevent PA such as walking, report a willingness to answer online questions and wear a PA monitor daily for 4 weeks, and come into the laboratory on two occasions (pre and post the 4 weeks of daily data collection). After the screening, eligible participants were scheduled for their first laboratory visit and were emailed a link to an online written consent form and baseline questionnaires (collected via SurveyMonkey^®^). Data were collected in two waves, the first wave (*n* = 10) occurred November-December 2014 and the second wave (*n* = 40) occurred February-March 2015. A total of 50 participants (36 who identified as women, 13 as men, and 1 as gender fluid) completed the phone screening, informed consent, and baseline questionnaires (90% Non-Hispanic White, 2% Asian-American, 4% Hispanic, 2% African-American, and 2% American Indian or Alaska Native). The average age reported was 39.36 years (range 21–73).

For wave 1, the first 10 participants to contact us were phone screened, deemed eligible, and entered into the study. Once we hit our target of 10 participants, the wave was closed and study staff removed all flyers. For wave 2, we again utilized flyers, listservs, and word of mouth (this included us asking wave 1 participants to tell others about the upcoming wave 2). Forty-three participants were screened, 3 were deemed ineligible, and we reached our target sample of 40 eligible participants for wave 2. For the 3 ineligibles, 1 was ineligible due to not being able to wear a Fitbit regularly as their worksite was a secure facility that did not allow for non-work devices. The other 2 were ineligible due to non-response after the screening (e.g., not showing up to the baseline session). As we neared our target of 40 participants for wave 2, study staff again removed all flyers to reduce the necessity of us having to turn away potential participants. An additional 11 people contacted us with interest in the study after wave 2 reached the target sample size. We thanked these people for their interest in our study, informed them that the study had reached capacity, and offered them the option of allowing us to contact them in the future for additional research.

### Procedure

Once participants were deemed eligible for the study via the phone screener (which took about 5 minutes), they scheduled an in-person session and were emailed a unique link to complete their online written informed consent and baseline questionnaires. After completing these online components (which always occurred prior to the in-person session), participants came to our lab for their pre-study in-person session. During this session, study staff reiterated the information on the consent form that participants had completed online (e.g., study procedure, compensation), answered any questions participants had, and measured the participants’ weight and height with a digital scale (Tanita BWB-800S; Tanita Corporation of American, Inc., Arlington Heights, IL) and stadiometer (Perspective Enterprises Standard Model). These measures were taken twice to ensure accuracy.

Next the lab staff gave participants the Fitbit device that they would be using and helped participants create an account on Fitbit.com. While it was not an exclusion criterion for the study, none of the participants had a preexisting Fitbit.com account. The objective weight and height measurements gathered in the lab were input into each participant's profile in order to calculate approximate daily calorie expenditures. Participants were asked to avoid modifications to weight, height, and passwords during the study process that were obtained at baseline. Participants were trained on using their Fitbit, including how to appropriately wear and charge the device, how to sync their data, and how to use the Fitbit application if they chose to do so on their mobile device. Participants were instructed to wear their Fitbit during all waking hours with the exception of any water-based activities (e.g., showering, swimming) as the device is not waterproof. Prior to the start of the study, all study personnel were given their own Fitbit and asked to use the device, set up (and use) an account at Fitbit.com, become familiar with navigating the website and mobile apps, and read through the FAQs on Fitbit’s website. Thus, study staff all gained expertise in utilizing the device, websites, and apps prior to their sessions with participants, and were well equipped to answer any questions that might arise.

Each participant’s in-person session was scheduled for 30 minutes to allow ample time for height and weight measurements and Fitbit training. Most participants’ sessions lasted 15–20 minutes. After training, staff scheduled the participant’s post-study in-person session (which would take place after the 4 weeks of data-collection), reminded participants of their research start date (11/20/2014 for wave 1, 02/09/2015 for wave 2), and informed participants on how to complete their online daily surveys. Participants were also provided with paper copies of the daily surveys in case of internet difficulties.

During the 4 weeks of data collection, participants received an automated daily email from the research team (email automated with Boomerang Gmail). Each email included a reminder to wear, charge, and sync their Fitbit, and a link to each daily diary survey (created with SurveyMonkey^®^), which participants were asked to complete immediately before going to bed (each daily survey was only open for a few hours during the evening/very early morning of the next day). Participants were also able to respond to the email and contact lab staff directly if they had questions or technical issues with the surveys or Fitbit devices. Emails were monitored daily so that issues could be dealt with quickly. Each week, lab staff would access SurveyMonkey^®^ to retrieve daily survey data and Fitbit.com to retrieve each participant’s PA data. At the end of the 4 weeks, participants completed the online follow-up questionnaires and returned to the lab for their post study appointment. During this visit the objective height and weight measurements were again taken twice using the stadiometer and digital scale, participants submitted any paper versions of daily surveys (if any were used), and received monetary compensation for participation. This visit usually took no more than 5 minutes.

Payment was given on a prorated basis: completing one of the four weeks resulted in a total of $5, two weeks $15, three weeks $30, and all four weeks $50 (a week was counted as complete if Fitbit and daily diary data were submitted for 5 out of 7 days). In addition, those who wore their activity monitor and submitted data for at least 21 of the 28 days were allowed to keep their Fitbit device. Finally, after the study was completed, participants were emailed a SurveyMonkey^®^ link to an anonymous survey soliciting feedback about their experiences.

### Measures

See S1 File for de-identified data for this study.

#### Retention

Participants were considered to have successfully been retained if they completed both in-person visits (the pre- and post- study sessions).

#### Adherence

For the daily diary surveys, adherence was the frequency of survey completion. This included both online surveys as well as paper versions of the survey that participants handed in (paper copies accounted for 1.4% of all completed surveys). For PA data, we focused on analyzing adherence in two ways. First, in line with past longitudinal studies utilizing Fitbit monitors [16, 17, 20], adherence was defined as the frequency of PA data available via the participants’ Fitbit.com account (that is any day with non-missing Fitbit data was considered a valid day). Second, we analyzed adherence by setting a minimum criterion for data to be considered valid. Specifically, we followed the protocol of past studies and set a Fitbit steps cutoff such that a day would be considered invalid if the total steps recorded for that day was below this number. Several different steps cutoffs have been used in the past including 300 steps [21], 1,000 steps [22], and 1,500 steps [23]. We opted for the 1,500 steps cutoff, as it was the most conservative of the three based on previous research [24].

Finally, we also explored adherence by examining the number of hours per day that steps were taken. This is a conservative method as participants could be wearing the Fitbit but not have steps data for that time (e.g., sitting, reclining) and thus hours of steps data is likely an underestimate of total wear time. This information was not in our original dataset as we had initially recorded the daily output from Fitbit (e.g., total steps taken that day) rather than hourly data. Thus, we sought and received approval from our Human Subjects Committee to re-contact participants and request permission to gather this information (e.g., participants changed their Fitbit.com password to one that we provided them with so we could access their data). These additional data were collected approximately 3 years after the initial study had ended. We were able to collect this data for 39 (78%) of our 50 participants. The remaining 11 participants did not respond (6 participants), could not be reached (e.g., phone number was no longer working; 3 participants), did not want to take part (1 participant), or were unable to help us (e.g., they had donated their device and no longer had access to the profile; 1 participant).

#### Participant feedback

Qualitative and quantitative feedback from participants were solicited post-study with an anonymous online survey. Participants were asked to assess 7 “How much did you like” items on a 5-point scale (ranging from *Really Disliked*, coded as -2, to *Neither Liked nor Disliked*, coded as 0, to *Really Liked*, coded as 2). These 7 items were “participating in the study,” “your Fitbit device,” “daily surveys,” “lab visits,” “baseline questionnaires,” “follow-up questionnaires,” and “Fitbit website.” Participants were also asked 5 open-ended questions: “What was your favorite part of the study?,” “What was your least favorite part of the study?,” “Any suggestions as to how we can improve the study?,” and two additional questions (relevant to the variables of interest for the overall study but not to this article) about PA and quality of life.

## Results

### Retention and adherence

**Fig 1:**
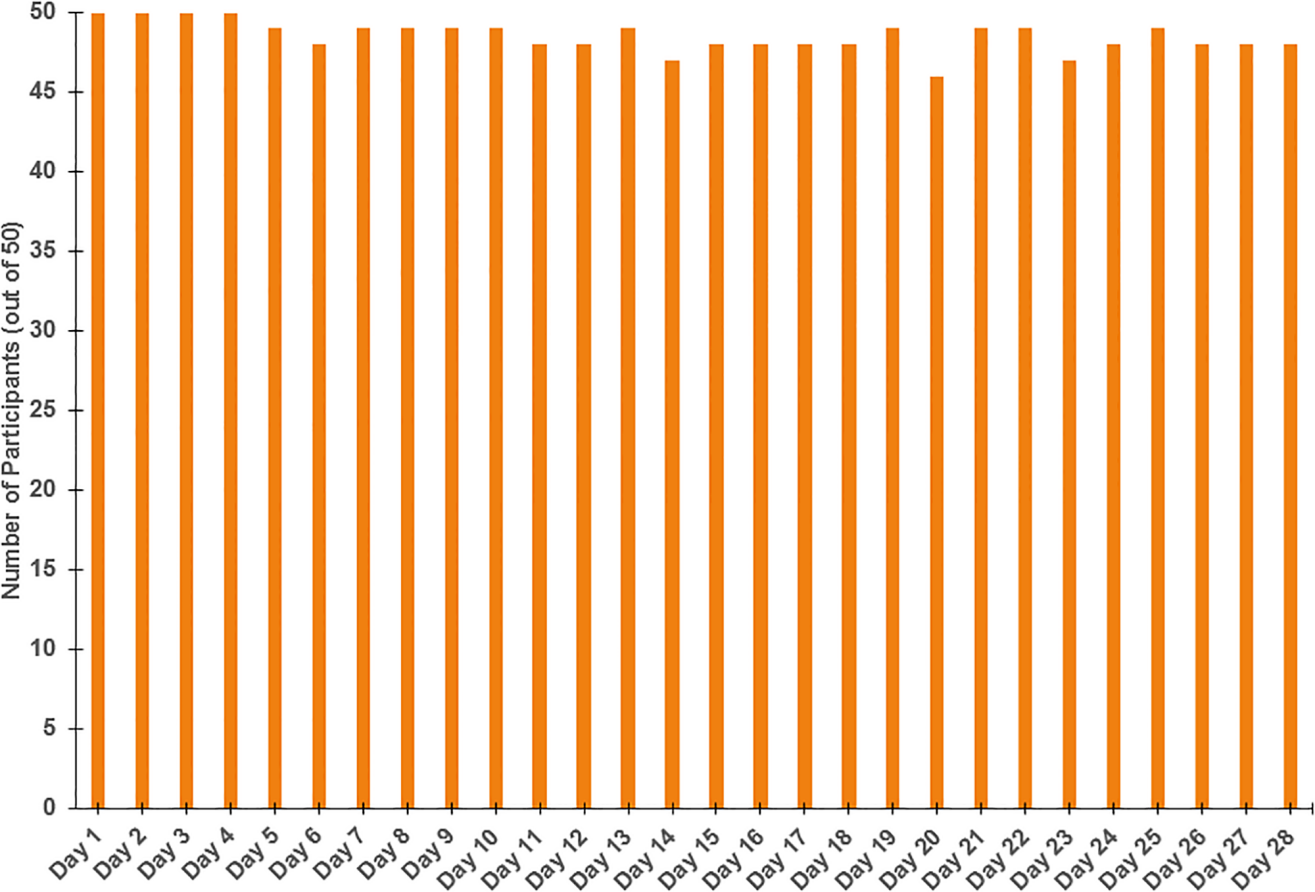
Daily questionnaires completed over 4 weeks across all participants.

Retention for this study was 100% with all participants completing both pre- and post-study lab in-person visits. For the surveys, an average of 48.50 participants (*SD* = 0.96; range 46–50) completed the daily survey on any given day of the 28 days, yielding 1,358 data points (97.00% adherence; see Fig 1). At the individual level, participants completed an average of 27.16 (*SD* = 3.27) questionnaires. There was one participant who only completed the daily questionnaires on 5 out of the 28 days. All other participants completed at least 25 out of the 28 daily questionnaires (*M* = 27.61, *SD* = 0.67). Overall, 34 participants (68%) completed all 28 questionnaires, another 12 (24%) completed 27 out of the 28, 2 (4%) completed 26 out of 28, 1 (2%) completed 25 out of 28, and 1 (2%) completed 5 out of 28.

**Fig 2:**
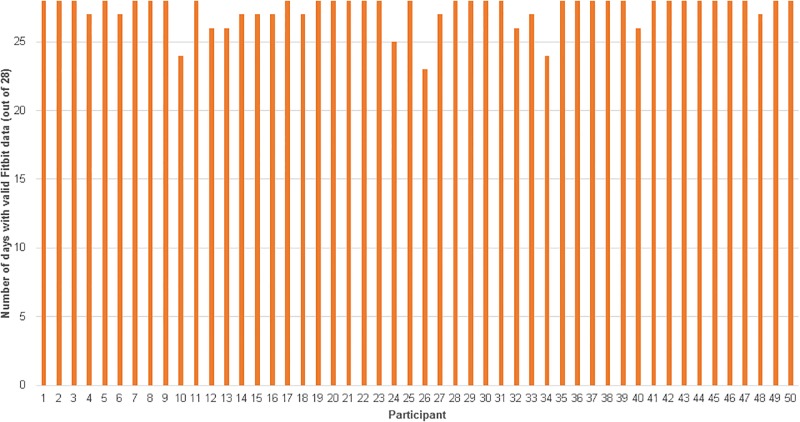
Adherence to wearing Fitbit device daily over 4 weeks.

For PA, an average of 49.79 participants (*SD* = 0.57, range 48–50) wore their Fitbit (for at least some of the day) on any given day of the 4 weeks, yielding 1,394 data points (99.57% adherence; see Fig 2). Participants wore their Fitbit on average for 27.88 (*SD* = 0.48, range 26–28) days. Forty-seven participants (94%) wore their Fitbit for all 28 days, while the remaining 3 participants (6%) wore their Fitbit for 26 days.

When using ≥1500 steps per day as the minimum criterion for a valid day, there were an additional 27 days out of the 1400 total (1.93%) that were invalid. Accounting for both missing and invalid data, there were 1,367 valid days of data (97.64% adherence; see Fig 2). Participants had on average 27.34 days (*SD* = 1.19, range 23–28) of valid Fitbit data. Thirty-three participants (66%) had valid Fitbit data for all 28 days. Forty-six participants (92%) had valid data for at least 26 out of the 28 days. Participants were generally active, averaging 8,738 steps per day (*SD* = 3,223) across all valid days with average daily steps ranging from 4,059 to 19,376.

Finally, we explored adherence via hours per day when steps were taken. We were able to recollect this data for 39 of our 50 original participants. For these 39 participants, steps were taken on average 14.99 hours each day (SD = 1.96, range 10.36–20.25). Using a typical criterion from Actigraph literature (at least 10 hours per day of wear-time) as a guide (e.g., [25, 26]), we flagged all days in which any participant had fewer than 10 hours of steps data. For 20 participants there were no flagged days, for the remaining 19 participants there were on average 1.46 flagged days (SD = 2.44, range 1–10). Overall, there were 57 total flagged days (94.79% adherence). We then examined the flagged days and (per [27]) looked for periods with no steps for 4 or more consecutive hours, using this as the criterion for invalid data. That is, if participants had steps data for <10 hours that day and more than one 4 (or more) hour gap in steps, then that day was deemed invalid (criterion set to more than one gap to allow for sleep). Using this criterion there were no invalid days for 24 participants and a mean of 1.13 invalid days (SD = 2.08, range = 1–7) for the remaining 15 participants. Overall, there were 44 invalid days (95.97% adherence).

### Participant feedback

Thirty-eight participants (76%) completed the post-study survey and provided anonymous feedback. Overall, in terms of the 7 quantitative items, participants reported liking all aspects of the study (see Table 1). Participants also shared a range of responses, mostly positive, for the qualitative items (see Table 2).

**Table 1:** Anonymous quantitative feedback about the study.

How much did you like/dislike:	Minimum	Maximum	Mean	SD
Participating in the study	0	2	1.34	0.67
The Fitbit device	-1	2	1.42	0.83
The daily surveys	-1	2	0.29	0.87
The lab visits	0	2	0.66	0.67
The baseline questionnaires	-1	2	0.47	0.65
The follow-up questionnaires	-1	2	0.49	0.69
The Fitbit website	0	2	1.29	0.61

Note: Scales ranged from -2 (Really Disliked) to 2 (Really Liked) with to 0 representing Neither Liked nor Disliked.

**Table 2:** Anonymous qualitative feedback about the study.

Favorite part of the study (*n*)	Least favorite part of the study (*n*)	Suggestions to improve the study (*n*)
Using the Fitbit/learning about PA activity (32)	Remembering to complete/completing the daily survey (24)	Some variant of “nothing” (17)
Fitbit/monetary incentives (5)	Remembering to wear/synch the Fitbit (7)	Shortening/varying daily survey or changing wording of specific items (10)
Motivating self to be more active (3)		Adding open-ended answer options to daily surveys (3)
Helping out research (1)		Using a different model of Fitbit or adding Fitbit app options (3)
		More flexibility for when daily surveys could be completed (2)
		Adding a practice day with the Fitbit before the study began (1)

## Discussion

### Strategies utilized to increase adherence

This study had high levels of retention and adherence, which we owe, in part, to our study staff. Study staff replied the same day to any participant questions/issues and throughout the research process (e.g., via email, during the phone screener, during in-person visits) and developed good rapport with participants by encouraging questions and explaining each step of the study in detail. Additionally, study staff emphasized the importance of wearing the Fitbit and completing the questionnaires consistently each day so that data would be of the highest quality and allow us to better understand the daily PA of typical adults (reminding participants not to change their behavior as this was not an intervention study). These interactions with the study staff likely positively influenced participation and the perceived value of the study by participants, as past research has shown that a spirit of collaboration between participants and researchers enhances compliance (e.g., [28]). In addition to staff characteristics and interactions, we utilized several additional strategies when preparing for and executing this study that helped contribute to these high numbers.

Firstly, we automated the data collection process as much as possible and automation allowed us to circumvent potential data collection issues. Automated daily reminders and questionnaires were sent via email (with embedded SurveyMonkey^®^ links) and PA data were automatically collected via Fitbit’s wireless synching. These automated reminders with embedded links (making it easy for participants to access and complete the surveys) have been shown to enhance response rates for online data collection (e.g., [15, 29]). Additionally, because one of the inclusion criteria was that participants were willing and comfortable with completing online questionnaires, we bypassed the issue of nonresponse due to unfamiliarity with computers, internet, and web surveys [15]. We also provided all participants at their pre-study visit with a packet of paper backups of all 28 daily surveys (in case online completion was not possible), although this accounted for only 1.4% of all completed surveys. Staff were trained to check the study email account regularly so that any participant issue could be dealt with quickly, and we kept a few additional Fitbit units in the lab in anticipation of potential loss or malfunction of participants’ units (this occurred for 2 participants, see Barriers and Issues section). This automation decreased both study staff and participant burden and minimized the chances that human error would negatively impact the data (e.g., if not automated, study staff could have forgotten to send out emails each day or participants could have forgotten to complete their paper survey if we did not utilize an online system). The daily surveys were also kept short (<5 minutes) to reduce participant burden and increase likelihood of responses (e.g., [29, 30]).

Secondly, we established a prorated incentive structure to enhance completion rates. Incentives have been shown to be effective in enhancing response rates, compliance, and retention. For example, a meta-analysis of mail response surveys found that prepaid monetary incentives increased response rates on average by 19.1%, with rates increasing as the incentives increased [31]. Another meta-analysis, focusing on interviewer-mediated surveys (e.g., face-to-face or via phone), found similar positive and linear effects of incentives with each dollar adding roughly 0.33% to the response rate [32]. In the clinical research domain, a review of studies of medication adherence found that incentives (most commonly monetary, although sometimes an alternative or additional incentive such as methadone, vouchers, or paid job training was utilized) increased compliance by an average of 20% [33]. These medication studies varied in terms of the dosing regimen (ranging from daily to once every 4 weeks) and duration of the study (4 weeks to 6 months). Monetary compensation also varied, ranging from $280 (for the 4-week study) to $4,800 (via paid job training across 24 weeks). For daily diary studies where maximizing participant responses to surveys is the desired outcome, monetary incentives tend to be modest. For example, Bolger, Zuckerman, and Kessler (2000) conducted a study of participants who were approaching a stressful life event (the bar exam) and their romantic partners, and offered $50 per couple for completion of the 35-day study [34]. The authors analyzed the first 32 days of data and utilized only the couples (68 out of the initial sample of 99) who completed the daily surveys at a very high rate (98%). For our study, because our timeline (28-days) and sample size (50) was similar to Bolger et al. (2000), we also utilized a monetary incentive of $50. However, we wanted to enhance adherence and retention and offer additional compensation beyond the $50 since we were asking participants to not only complete a daily survey but also wear a Fitbit. Thus, our incentive structure was $5 for completing 1 week, $15 for 2 weeks, $30 for 3 weeks, and $50 for all 4 weeks. Participants were also allowed to keep their Fitbit if they provided at least 3 weeks (75%) of both daily diary and Fitbit data. While this incentive structure was designed to enhance retention and adherence, it is notable that no participant dropped out of the study or stopped providing data after 3 weeks (when they “earned” the Fitbit device). Similarly, even though participants could have earned full compensation by providing data on 5 out of 7 days each week (our threshold for a “complete” week of data), the vast majority of participants completed all 7 days of data for all 4 weeks. That is, a participant could have maximized compensation while minimizing reporting by wearing the Fitbit and responding to surveys on 20 out of the 28 days; however, participants on average wore their Fitbit on 27.88 days and responded to the surveys on 27.16 days. Additionally, on the anonymous feedback, 32 participants reported that their favorite aspect of the study was using the Fitbit and learning about their own PA, while only 5 reported on the Fitbit/monetary incentive. This feedback also indicated that participants had a strong intrinsic motivation (e.g., learning about their own behavior) for completing the study, which is in line with research suggesting that interest and curiosity are positively related to research participation and response rates (e.g., [30, 35]). Furthermore, anecdotally, several participants expressed surprise at the monetary compensation during the post-study session (having forgotten about this component), and one participant expressed no interest in the compensation, completing all assessments but needing several prompts (and even suggesting that we keep the money) before being convinced to come in and receive payment. Taken together, these findings suggest that motivations beyond the incentives were also present in this study.

Finally, we collected data in two waves and emphasized to participants the importance of their adherence and participation—that the study and their efforts would help us better understand PA. This emphasis helped to establish a spirit of collaboration wherein the participants were an important partner in the generation of scientific knowledge and useful information could benefit the public, something that past research has shown to enhance compliance (e.g., [28, 36]). Participants also typically valued science/research (consistent with literature that links this valuing to participation, e.g., [35]), asking many questions, exhibiting a desire to understand and comply with the study instructions and to provide high-quality data, and expressing interest in future research (i.e., 92% of participants responded on the follow-up questionnaire that we could contact them for other studies). This two-wave system also allowed capitalization on the enthusiasm of our participants, as they became an important recruitment tool (via word of mouth) for wave 2. Potentially, wave 1 participants also referred others who were similarly interested in assisting research and conscientious into our wave 2. The two-wave process (recommended by [15]) also allowed us to utilize wave 1 as a sort of pilot and solicit feedback from participants for any tweaks that needed to be made to the study protocol or survey content. The feedback was very positive from wave 1, and thus we were more confident going into wave 2 that our protocol was palatable to participants.

### Barriers, issues, and lessons learned

Overall the study ran smoothly with few issues. The main difficulty, inherent to studies with rigid timelines, was scheduling participants to come into the lab as close as possible to the start- and end-date of the 4-week study. In anticipation of this issue, the timeline was emphasized during recruitment and we utilized the start-date as an exclusion criteria during screening (i.e., those who were not able to come in prior to the start-date or who were not able to begin the study on the start-date were deemed ineligible). Additionally, we had dedicated staff with flexible availabilities which allowed for laboratory sessions that accommodated participants’ schedules (e.g., evenings and weekends). Two of the 50 participants lost their Fitbit during the study and notified us of the loss when it occurred. We had anticipated that Fitbits may need to be replaced due to loss, damage, or technical issues, and had additional units available. We were able to provide replacements quickly (as soon as participants notified us of the loss we scheduled a time to provide them with another unit) and thus minimized data loss. The lost Fitbits represented 2 full days of lost data for 1 participant and 2 full days and a partial day of lost data for the other (all 5 days were treated as missing/non-adherent when using the ≥1500 steps for a valid day rule). Finally, 6 participants experienced issues with synching the Fitbit. We communicated with these participants quickly to resolve the issues. After the first incidence of this issue, we compiled a brief troubleshooting guide that we later utilized to streamline the process.

In hindsight, we realized that there were additional data that we could have collected during the study that would have been helpful to our understanding of Fitbit adherence. While the two main methods that we used (any wear and steps of at least 1,500 per day) are common in the Fitbit literature, additional data such as hours per day with steps could have been collected. Collecting these data at the time of the study would have allowed us to have full hourly data for all participants and preempted the need to contact participants years later. Additionally, we could have more directly measured Fitbit wear-time via self-report as has been done in some past studies [23, 37–38]. The addition of explicit wear-time information would have allowed us to utilize the minimum 10 hours per day criterion from the Actigraph research literature. This criterion would have been a more nuanced measure of adherence than whether or not the device was worn, and potentially a more accurate adherence measure than the 1,500 steps per day cutoff.

## Conclusions

Daily repeated measurements provide much richer data to scientists than cross-sectional designs. These designs are especially relevant to sports medicine researchers who may be interested in daily physical activity and other related variables, particularly in the context of the many monitors and other equipment available for measuring objective activity. However, missing data from low response rates and attrition are problems that daily response studies must adequately address for results to be meaningful. This paper presented adherence and retention data from a 4-week daily diary study. There were few missing data points for the 50 participants across the 28-days. The results show the feasibility of conducting a 4-week daily diary study utilizing self-report and PA monitor data collection. This paper also provided evidence of effective strategies for enhancing adherence and retention for both self-report and PA monitor components of a 4-week daily assessment study. These strategies included automation of data collection, having dedicated and responsive study staff who had strong rapport with participants, emphasizing the importance of participant adherence, providing incentives for retention and adherence, utilizing 2 waves of data collection and asking for participants’ help in recruitment for the second wave, and anticipating potential barriers and issues and taking steps to prepare for these.

From our lessons learned, we recommend that researchers consider wear-time prior to data collection and adjust their methodology accordingly. For example, researchers could add a daily self-report measure of wear-time to their study. For objective wear-time, researchers could use devices such as Actigraphs where wear-time can be inferred from specific movement data that are recorded (this cannot be done with the Fitbit One as motion other than steps are not automatically recorded and thus sedentary time is indistinguishable from non-wear time). Researchers could also consider using later models of Fitbit that record heart rate data, as these data should be present even when participants are sedentary, allowing researchers to infer that the device is being worn (although researchers should be aware that there may be few or no published validation studies on newer models of monitors).

We hope that the strategies presented in this paper and discussion of barriers/issues as well as how these were dealt with will be helpful to other researchers designing repeated-measures studies. However, we are aware that every research study is idiosyncratic and many factors (e.g., study personnel, participant variables, assessment method and content, etc.) influence participant compliance and retention. For example, there may have been factors specific to our particular sample (e.g., sample characteristics from this part of the country) that helped with retention and adherence, a potential area of inquiry for future studies. The points presented in this paper may be useful to health researchers interested in longitudinal data collection; however, further research with longer durations, larger and more diverse samples, and within intervention frameworks are needed to verify the general applicability of these strategies.

## Supporting information

S1 File De-identified dataset. (XLSX)
